# Fast Alpha Activity in EEG of Patients With Alzheimer’s Disease Is Paralleled by Changes in Cognition and Cholinergic Markers During Encapsulated Cell Biodelivery of Nerve Growth Factor

**DOI:** 10.3389/fnagi.2022.756687

**Published:** 2022-04-25

**Authors:** Helga Eyjolfsdottir, Thomas Koenig, Azadeh Karami, Per Almqvist, Göran Lind, Bengt Linderoth, Lars Wahlberg, Åke Seiger, Taher Darreh-Shori, Maria Eriksdotter, Vesna Jelic

**Affiliations:** ^1^Department of Neurobiology, Care Science and Society, Karolinska Institutet, Solna, Sweden; ^2^Theme Inflammation and Aging, Karolinska University, Stockholm, Sweden; ^3^Translational Research Center, University Hospital of Psychiatry, University of Bern, Bern, Switzerland; ^4^Department of Clinical Neuroscience, Stockholm, Sweden; ^5^Department of Neurosurgery, Theme Neuro, Karolinska University, Stockholm, Sweden; ^6^NsGene Inc., Providence, RI, United States; ^7^Stiftelsen Stockholms Sjukhem, Stockholm, Sweden

**Keywords:** EEG, EEG alpha activity, Alzheimer’s disease, nerve growth factor, encapsulated cell biodelivery, choline acetyltransferase

## Abstract

**Background:**

Basal forebrain cholinergic neurons are dependent on nerve growth factor (NGF) for growth and survival and these cells are among the first to degenerate in Alzheimer’s disease (AD). Targeted delivery of NGF has been suggested as a potential therapy for AD. This hypothesis was tested in a clinical trial with encapsulated cell biodelivery of NGF (NGF-ECB) in AD patients. Three of six patients showed improved biomarkers for cognition by the end of the study. Here, we report on the effects of targeted delivery of NGF on human resting EEG.

**Materials and methods:**

NGF-ECB implants were implanted bilaterally in the basal forebrain of six AD patients for 12 months. EEG recordings and quantitative analysis were performed at baseline, 3 and 12 months of NGF delivery, and analyzed for correlation with changes in Mini-mental state examination (MMSE) and levels of the cholinergic marker choline acetyltransferase (ChAT) in cerebrospinal fluid (CSF).

**Results:**

We found significant correlations between the topographic variance of EEG spectral power at the three study points (baseline, 3 and 12 months) and changes in MMSE and CSF ChAT. This possible effect of NGF was identified in a narrow window of alpha frequency 10–11.5 Hz, where a stabilization in MMSE score during treatment was related to an increase in EEG alpha power. A similar relation was observed between the alpha power and ChAT. More theta power at 6.5 Hz was on the contrary associated with a decrease in CSF ChAT during the trial period.

**Conclusion:**

In this exploratory study, there was a positive correlative pattern between physiological high-frequency alpha activity and stabilization in MMSE and increase in CSF ChAT in AD patients receiving targeted delivery of NGF to the cholinergic basal forebrain.

## Introduction

Resting-state quantitative EEG (qEEG) is a good candidate marker for intervention clinical trials in Alzheimer’s disease (AD) since it has shown good discriminatory power between AD patients and healthy aging in several studies (Brenner et al., 1988; Lehmann et al., 2007; Jelic and Kowalski, 2009; Babiloni et al., 2021). These studies report on an increase in the slow frequency activities including early changes in theta range and the later stage of the disease alterations in the delta range, in parallel with a decrease in the faster alpha and beta frequencies.

Already at the very beginning of acetylcholine esterase inhibitor (ChEI) treatment for AD, it was reported that chronic treatment with cholinergic drugs induces a specific pattern of electrical brain activity corresponding to both the treatment response and early decline in treatment efficacy (Jelic et al., 1998).

It has been shown repeatedly that the ChEI treatment increases the physiological alpha rhythm oscillations in EEG, particularly in the posterior regions (Jelic et al., 1998; Moretti, 2014). Furthermore, these positive effects on the resting EEG spectral alpha power induced by ChEIs, correlated well with improved cognitive function as measured by significant changes in Mini-mental state examination (MMSE) scores (Moretti, 2014). Recently an EEG-based acetylcholine (ACh) index has been developed using data from a scopolamine challenge study showing a reduced ACh index even in prodromal AD (Johannsson et al., 2015). Interestingly, even cholinergic stimulation of healthy subjects with the ChEI galantamine showed an effect on the alpha power and working memory performance (Eckart et al., 2016).

The symptomatic cholinergic treatment approach to AD is based on findings that Nucleus Basalis of Meynert (NBM) is a major source of cholinergic projections to the cortex (Mesulam and Geula, 1988) and reductions in levels of the cholinergic markers acetylcholinesterase (AChE) and choline acetyltransferase (ChAT) reflects the loss of cholinergic innervation that have been reported in the AD brain (Giacobini, 2003). Traditionally, ChAT activity is used as a marker of cholinergic neuronal loss in lesion studies (Wenk et al., 1994; Rossner et al., 1995). It has been shown that NBM lesions in animal models decrease cortical ChAT activity and modulate event-related oscillations in EEG by increasing power in low and decreasing power in high frequencies (Sanchez-Alavez et al., 2014).

Nerve growth factor (NGF) has emerged as a potential therapeutic agent in Alzheimer’s disease (AD) due to its regenerative effects on basal forebrain cholinergic neurons (Williams et al., 2006; Mufson et al., 2008). Exogenous NGF delivered to the cholinergic basal forebrain has shown regenerative effects correlating with improved cognition in animal models of AD (Hefti and Mash, 1989; Olson et al., 1992). Since NGF does not cross the blood-brain barrier, administration to the brain provides a challenge.

Nerve growth factor administration to the brain has previously been tested in clinical studies of AD patients. A first trial was performed in three AD patients where NGF was delivered by intracerebroventricular infusion (Eriksdotter Jönhagen et al., 1998). The results indicated an up-regulation of nicotinic receptors and glucose metabolism on positron emission tomography (PET), as well as a normalization of the electroencephalography (EEG) pattern. However, adverse effects of NGF (neuropathic pain being the most prominent), made this route of administration unacceptable for routine treatment. In a clinical trial using genetically modified fibroblasts secreting NGF injected into the basal forebrain in AD patients, Tuszynski et al. (2005) demonstrated the feasibility of an *ex vivo* gene therapy approach with results indicating a slowing of disease progression. Our group has shown that targeted delivery of NGF through encapsulated cell biodelivery (NGF-ECB) into the basal forebrain is safe and well-tolerated (Eriksdotter-Jönhagen et al., 2012; Wahlberg et al., 2012). Three of the six patients responded to the NGF-delivery with a decrease in brain atrophy (Ferreira et al., 2015), and an increase in cholinergic markers in cerebrospinal fluid (CSF), correlating with improved cognition and brain glucose metabolism (Karami et al., 2015). Importantly, the activity of the acetylcholine synthesizing enzyme, ChAT in CSF showed a significant increase in patients with stable cognition during the 12-month NGF delivery (responders), compared to those patients who declined cognitively (non-responders) (Koenig et al., 2011).

The present report aimed to explore the potential effects of NGF-ECB administration on qEEG parameters during a 12-month exploratory study in a small, but unique sample of AD patients that have undergone this advanced experimental treatment. Our findings corroborate the previously reported changes in the rate of brain atrophy (Ferreira et al., 2015) and cholinergic markers in CSF (Karami et al., 2015) and further investigate changes in the functional measure of synaptic brain activity such as EEG. Moreover, we wanted to investigate whether qEEG changes were related to the global measures of cognitive stabilization and the levels of cholinergic markers in the CSF by using an advanced statistical method to analyze multichannel EEG data.

## Subjects and Methods

### Participants, Clinical Assessments, Procedures, and Ethics

The details of the NGF-ECB study as well as patient demographic and clinical data have been previously reported (Eriksdotter-Jönhagen et al., 2012; Wahlberg et al., 2012). In brief, six patients with mild to moderate AD, the median age of 62 years (range 55–73), and a median MMSE score of 23 (range 19–24) were enrolled and completed the study. They were all on stable ChEI treatment at baseline (median of 12 months, range 8–26 months) and throughout the study.

Before enrollment, the patients underwent a comprehensive medical evaluation, including medical history, somatic assessment and cognitive screening with MMSE (Folstein et al., 1975), lumbar puncture for CSF analyses, computerized tomography (CT) or magnetic resonance imaging (MRI), psychometric testing of cognition, EEG and routine blood sampling. All mentioned procedures were repeated within 1 week at 3 and 12 months follow-up. AD diagnosis was confirmed histopathologically on a cortical biopsy from surgical implantation (Vijayaraghavan et al., 2013).

The NGF-ECB implant is a catheter-like device containing a human retinal epithelial cell line, genetically modified to secrete NGF. All six patients received bilateral single implants in the Ch4 region in the basal forebrain and the last three patients also received additional bilateral implants in the Ch2 region as previously described (Rosengren et al., 1996; Vijayaraghavan et al., 2013). None of the patients suffered complications related to the neurosurgery or the device, and the post-operative courses were mainly uneventful (Rosengren et al., 1996; Vijayaraghavan et al., 2013). All patients completed the study, including the removal of all implants after the 12-month study period.

The patients were monitored primarily for safety and tolerability. Secondary outcome measures included effects on cognition, imaging, and quantitative EEG (qEEG) parameters, the latter being an additional exploratory outcome.

The study was conducted according to the Helsinki Declaration and was approved by the Swedish Medical Products Agency. Ethical approval was obtained from the Regional Human Ethics Committee of Stockholm. Both patients and caregivers gave written informed consent before study entry.

### EEG Recordings

All spontaneous EEGs were recorded in a resting awake condition during the morning between 8 and 12 p.m. using the Nervus digital recording system (Natus Medical/NicoletOne). Electrodes were placed according to the standard 10/20 system, the reference electrode was placed in the midline between Fz and Cz, and the ground electrode between Cz and Pz. Horizontal and vertical eye movements and blinking were monitored by an EOG channel. Electrode impedance was below 5 kΩ. Initial filter settings were: low pass online filter 70 Hz to de-noise various types of interferences during the recording and sampling rate 256 Hz. The patients were seated in a slightly reclined chair in a sound-attenuated, normally lit room and after completion of the EEG set-up were instructed to remain relaxed, yet alert and awake during recording. Trained biomedical engineers were continuously monitoring the level of consciousness and used acoustic stimulation (noise or calling a patient’s name) to keep the patients awake during the recording. Duration of resting-state eyes-closed EEG recording was 15 min with intermittent 5 s eyes open intervals during the first 10 min of recording, followed by 5 min eyes closed recording.

### Quantitative EEG Analysis

EEGs were exported in an average reference mode into an EDF-file format and computerized EEG analysis was performed offline using the commercially available software, Brain Vision Analyzer, version 2.1 (Brain Products GmbH, Gilching, Germany). Non-physiological and physiological artifacts due to eye movements, contamination with muscle activity, and episodes with drowsiness were removed after visual inspection of the recording, and only segments with the awake eyes-closed state during the recording were included in the analyses. Ocular artifacts were additionally removed from all channels by using EOG and additionally semi-automated independent component (ICA) algorithm. Drowsiness during EEG recording was defined by transients of slow rolling eye movements (SREM) and concomitant attenuation of posterior alpha rhythm or occurrence of frontocentral theta activity. The preprocessed EEG data were segmented in 2-s epochs, frequency transformed at all electrodes using Fast Fourier Transform (FFT) algorithm with a Hanning window which is an in-built solution in the Brain Vision Analyzer software used to smooth out the endpoints of the data before applying FFT and thus reduce spectral leakage. All frequency transformed epochs were thereafter averaged to yield one amplitude/power spectrum per patient.

### Cerebrospinal Fluid Biomarkers

Cerebrospinal fluid samples were collected by lumbar puncture before implantation (at baseline), 3 and 12 months after implantation. The samples were aliquoted and kept in polypropylene tubes at −80°C until analyzed. CSF ChAT activity was measured by a colorimetric assay as previously described (Vijayaraghavan et al., 2013). CSF levels of neurofilament light chain protein (NFL) were analyzed using a previously described enzyme-linked immunosorbent assay (ELISA) method (Rosengren et al., 1996).

### Statistical Analyses

The analysis of treatment effects on the frequency domain EEG scalp distribution was based on topographical analyses of covariance (TANCOVAs) (Koenig et al., 2008), testing whether a statistically significant amount of topographic variance can be accounted for by the linear contribution of an individual external predictor that may vary by condition, and there the significance of such accounts is assessed globally across all electrodes using a randomization test. For the testing of EEG associations with an external predictor that interacts with some condition, first, electrode-wise regression maps with the external regressor are computed. The difference of these regression maps is then globally quantified by first subtracting, from all regression maps the mean regression map, and then computing the overall mean square of the residual. To estimate the distribution of this RMS measure under the null hypothesis, the procedure is then also applied to data where the condition and subject labels have been randomly permutated. The statistical significance is given by the percent rank of the original measure in comparison to the estimated distribution under the null hypothesis. TANCOVAs have the advantage that they do not require any *a priori* hypothesis about a particular spatial distribution of some effect, which limits type I errors, but need, for a given frequency, only one test for the entire set of electrodes, which strongly reduces type II errors. This is particularly useful in the current context, where a larger number of subjects, and thus more statistical power, is not possible due to the costs and the invasive nature of the study. For the computation of the TANCOVAs and the visualization of the results, we used a Matlab-based program, Ragu (Randomization Graphical User interface) (Koenig et al., 2011), and estimated null-hypothesis based on 1,000 randomization runs. TANCOVA uses non-parametric randomization statistics that do not require a Gaussian distribution of the variable values across subjects.

No correction for multiple comparisons was used since randomization tests use global measures of effect size and therefore “downsize” a large amount of data by using a single measure of effect size which makes it suitable for this type of exploratory analysis. This results in a significant reduction of the tests needed for comparisons which in return, avoids correction of the results for multiple comparisons (Gudmundsson et al., 2007).

In the present analysis, MMSE and ChAT were considered as external predictors and the EEG spectral power maps at baseline, after 3 and 12 months of NGF delivery were considered as repeated measures. As this was an interventional study, we were primarily interested in systematic EEG covariates of the interaction of clinical measures with time after the intervention, which was computed for each frequency bin in the range from 1 to 30 Hz. Frequency bins are intervals between points in frequency-transformed data and thus bins refer to each 0.5 Hz frequency point computed separately for 1–30 Hz spectral range.

## Results

Six patients were enrolled in the study, two men and four women, implanted with NGF-ECB implants in the basal forebrain for 12 months, when the implants were removed uneventfully. All six patients were treated with ChEI as concomitant medication for a median duration of 12 months (range 8–26) before enrollment and continued this therapy throughout the study (Table 1).

**TABLE 1:** Demographic data on enrolled patients.

	Patients
Gender, *n* (M/F)	4 M/2 F
Age, median (range)	62 (55–73)
Memory problems (y), median (range)	4 (1–6)
AD diagnosis (y), median (range)	1.5 (1–3)
Duration of ChEI (m), median (range)	12 (8–26)
MMSE score at baseline, median (range) ● Non-responders ● Responders	23 (19–24) 23 (19–24) 23 (21–23)
MMSE score at end of study, median (range) ● Non-responders ● Responders	18 (14–27) 15 (14–16) 21 (21–27)
CSF ChAT activity (nmol/min/ml), baseline, median (range) ● Non-responders ● Responders	2.6 (2.0–3.7) 2.7 (2.0–3.4) 2.6 (2.6–3.7)
CSF ChAT activity (nmol/min/ml), 3 months, median (range) ● Non-responders ● Responders	2.7 (2.3–3.1) 2.7 (2.3–2.7) 3.1 (2.5–3.1)
CSF ChAT activity (nmol/min/ml), 12 months, median (range) ● Non-responders ● Responders	3.0 (2.0–4.3) 2.9 (2.0–3.0) 3.7 (3.0–4.3)
CSF NFL (ng/L), baseline, median (range)	157.5 (125–360)
CSF NFL (ng/L), 12 months, median (range)	260 (125–420)

*AD, Alzheimer’s disease, ChAT, choline acetyltransferase, ChEI, cholinesterase inhibitors, CSF, cerebrospinal fluid, F, female, M, male, MMSE, Mini-mental state examination, NFL, neurofilament light protein.*

### Association Between EEG Spectral Power and Cognitive Stabilization During Nerve Growth Factor Delivery

**FIGURE 1:**
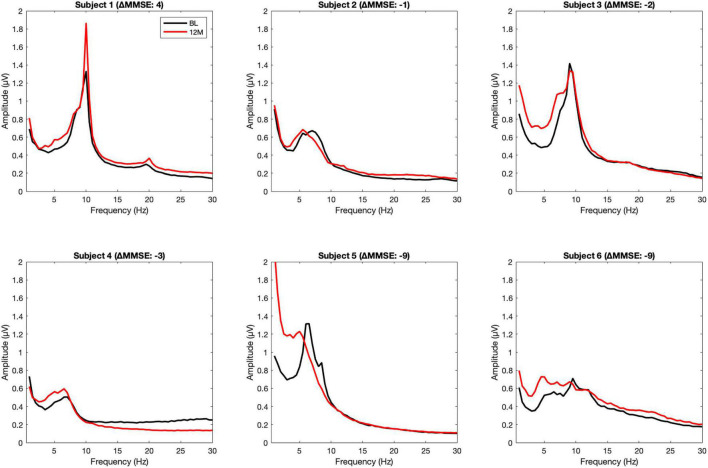
Averaged power spectra, baseline vs. 12 months, per individual subject. The upper row shows the power spectra of responders, the lower row of the non-responders.

We tested the data for significant changes in EEG spectral power in the frequency range from 1 to 30 Hz and if there were associations found with MMSE change at 12 months of NGF delivery against a baseline, as a function of time of the EEG recording (baseline, 3 months or 12 months). Figure 1 illustrates change in averaged power spectra, baseline vs. 12 months, per individual subject.

**FIGURE 2:**
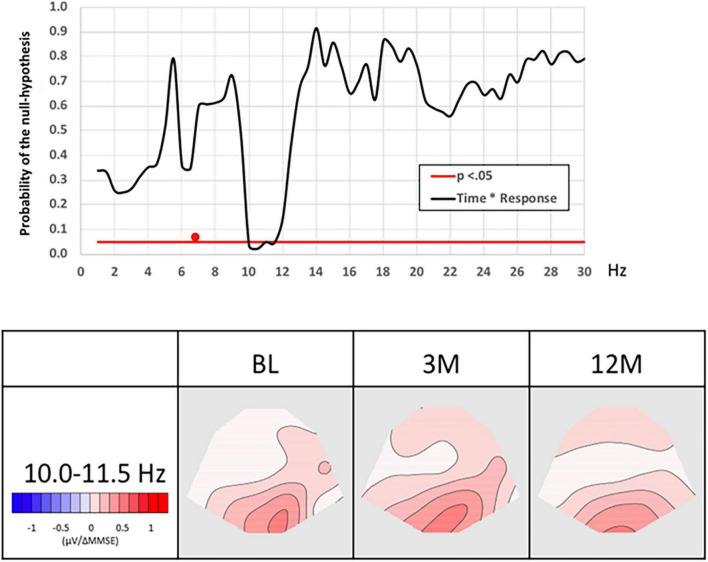
Association between MMSE and EEG spectral power as a function of time. The graph on the left the probability (p) of the null hypothesis (*y*-axis) for the TANCOVA interaction between time and MMSE change as a function of frequency in Hz (*x*-axis). Significant (*p* < 0.05) frequency range is marked by a white line shows. The red cursor marks the frequency range represented by the covariance maps. Topographic maps display topographic covariance of the EEG spectral power correlating to MMSE changes at the **frequency range 10–11.5 Hz** for the three EEG recording occasions. Red maps show were less than the average change in MMSE score related to an increase in alpha frequency. BL, baseline; EEG, electroencephalography; MMSE, mini-mental state examination; TANCOVA, topographic analysis of covariance, 3 m: 3 months, 12 m: 12 months.

The interaction between EEG frequency spectra across the three recordings and two groups, based on above or below the mean change in MMSE (12 months minus baseline), for the six patients showed a significant association at around 11 Hz (10–11.5 Hz), *p* = 0.042, Figure 2. *Post hoc* tests: baseline against 3 months *p* = 0.059, the baseline against 12 months *p* = 0.033, the baseline against merged 3 and 12 months *p* = 0.023, 3 months against 12 months, *p* = 0.56. This suggests a correlation between change in MMSE and EEG activity in the narrow window of the upper alpha band that was marginally significant at 3-months and significant at 12-months of NGF delivery. The fact that there was no significant correlation between the 3 and 12 months implicates that most clinically detectable change happens in the first 3 months of treatment. The effect remains sustained during the treatment, although no further increase is detectable between the 3 and 12 months. No significant effects were observed when the data were merged across all observational points.

### Association Between Changes in Cerebrospinal Fluid Choline Acetyltransferase Activity and EEG Spectral Power

**FIGURE 3:**
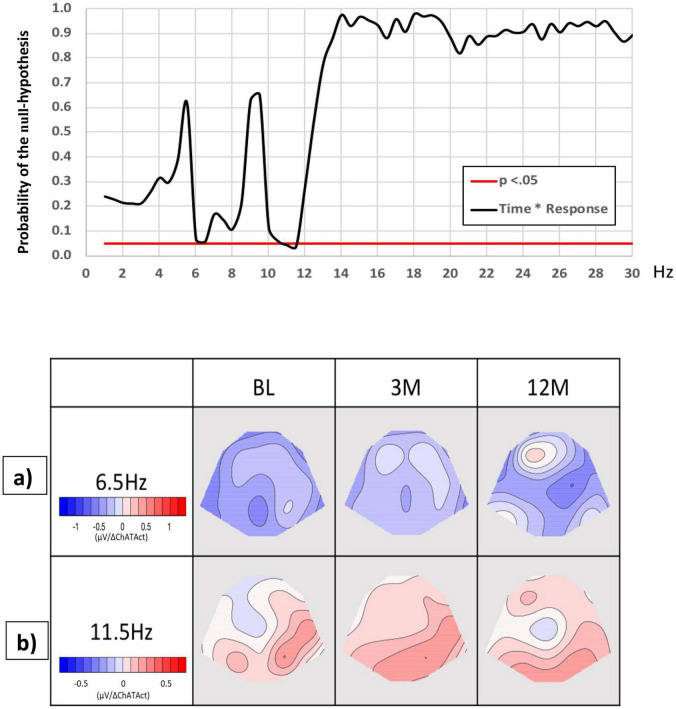
Associations between CSF ChAT activity and EEG spectral power as a function of time during the NGF delivery. The graph shows the probability (p) of the null hypothesis (*y*-axis) for the TANCOVA interaction between time and change in CSF ChAT activity, as a function of EEG frequency in Hz. The significant (*p* < 0.05) frequency range is marked by a white line. The red cursor marks the frequency range represented by the covariance maps. Maps display topographic covariance of the EEG spectral power correlating to changes in ChAT activity at the frequency ranges: (a) **6.0–6.5** for the three EEG recording occasions. The blue maps show where lower than the average difference in CSF ChAT values correlated with an increase in the theta frequency, (b) **11.0–11.5 Hz** for the three EEG recording occasions. The red maps indicate where a higher than average difference in CSF ChAT activity (12 months vs. baseline) is related to an increase in upper alpha frequency. BL, baseline; ChAT, choline acetyltransferase; CSF, cerebrospinal fluid; EEG, electroencephalography; TANCOVA, topographic analysis of covariance, 3 m: 3 months, 12 m: 12 months.

TANCOVA correlations between EEG frequency spectra across the three recording occasions, dividing the six patients into two groups according to a change in CSF ChAT activity, below or above the mean change (12 months minus baseline), showed a marginally significant correlation in the frequency of around 6.0–6.5 Hz, Figure 3. This indicates that the more power in the theta frequency band, the less the change in CSF ChAT activity, *p* = 0.058 (Figure 3a). *Post hoc* tests: baseline vs. 3 months (*p* = 0.09), baseline vs. 12 months (*p* = 0.06), 3 months vs. 12 months (*p* = 0.29), baseline vs. 3 and 12 months merged (*p* = 0.023). The results suggest that the relationship between a reduction in CSF ChAT activity and EEG slowing in a narrow theta frequency range was mostly detectable at 3 months.

We also found a significant correlation between time vs. change in ChAT activity at 11–11.5 Hz (*p* = 0.025), Figure 3b. The interaction was mainly accounted for by the difference between baseline and 3 months, indicating that at 3 months of NGF delivery, the patients showed more alpha power correlating to positive changes in CSF ChAT activity, compared to baseline. Hence, the increase in ChAT seems to predict an increase in fast alpha activity up to 3 months of treatment. *Post hoc* tests: baseline vs. 3 months (*p* = 0.042), baseline vs. 12 months (*p* = 0.21), 3 months against 12 months (*p* = 0.12), baseline against merged 3 and 12 months (*p* = 0.056).

### Association Between Changes in Cerebrospinal Fluid Nerve Growth Factor and EEG Spectral Power

There was no correlation between changes in NFL and EEG at any time point, and no significant interaction with time, MMSE, and ChAT.

## Discussion

This exploratory study is the first to report a correlation between the cholinergic marker ChAT in CSF and EEG oscillations in the alpha and theta frequency range during encapsulated NGF delivery to patients with a definite AD diagnosis as confirmed by a cortical biopsy (Eriksdotter-Jönhagen et al., 2012). Associations between changes in ChAT activity and EEG in narrow windows of both the theta and alpha frequency bands were mainly observed during the first 3 months of NGF delivery and do not seem to increase further thereafter. These limited effects could be explained by the fact that all the patients already were on a stable long-term treatment with ChEI which is a requirement for all clinical trials with new experimental treatments.

This was also the first cohort of patients who received NGF delivery *via* the NGF-ECB implants, with safety and tolerability as primary endpoints. However, at implant retrieval at 12 months, the NGF release was low (Eriksdotter-Jönhagen et al., 2012). It would be of interest to study the delivery of NGF with NGF-ECB implants for a longer duration than 12 months at sustained NGF dose level, to see if there are similar or even more pronounced effects on EEG, cholinergic biomarkers, and cognition.

In our study, a cognitive stabilization in some patients, as measured by an increased or stable MMSE score during the NGF delivery was related to a significant effect in a narrow frequency of the fast alpha band 10–11.5 Hz. Although such a narrow window of significant correlations might be the result of small sample size and could be questioned as physiologically relevant, an alternative explanation for the selective effect on the fast alpha frequencies may be the different functional significance of slow and fast alpha oscillations (Klimesch, 2012). It has been suggested that activities in the lower alpha bands (slower alpha oscillatory frequencies) are related to attentional processes and that those of the higher alpha bands (faster alpha oscillatory frequencies) are related to retrieval from the long-term memory (Klimesch, 1996). Although this knowledge is generated in EEG studies during a cognitive activation paradigm, we could speculate that it could be extrapolated to the resting EEG during stimulation of the cholinergic system, which may preferentially activate neuronal networks involved in selective cognitive processes.

An alternative explanation may be that both thalamocortical and cortico-cortical networks are responsible for the generation of rhythmic alpha oscillations, assuming that the balance with other neurotransmitter systems during the disease process in AD affects the profile of EEG changes and cognitive response (Dringenberg, 2000).

While our earlier study showed that treatment with ChEI gave limited improvements of qEEG parameters up to 6 months (Jelic et al., 1998), this study showed a sustained association between positive changes in qEEG profile in the patients who showed clinical stabilization as suggested by MMSE change after a 12 month NGF delivery. The narrow windows of correlations between the EEG frequency spectra and cognitive and biochemical markers of cognitive deterioration and biological activity of the disease during the NGF-ECB treatment should be interpreted with a reservation as a converging pattern in the context of current knowledge.

There was no strong consistent relation with theta power and the MMSE change as was shown for alpha power. Theta activity is known to increase early during AD and reaches a plateau during the disease course (Musaeus et al., 2018). Thus, theta power may not be a single sensitive electrophysiological state marker for monitoring disease activity and effects of therapeutic stimulation of the cholinergic system but can rather be considered an indicator of the baseline disease severity and could therefore be a basis for the selection of patients for clinical trials.

One limitation of this study is the small number of patients, which increases the variance of the data and puts limits to the use of conventional statistics. We propose here the use of the Ragu program which by using assumption-free randomization statistics computes significance as a function of frequency, controls for type 2 error, and displays results in a user-friendly visual format with EEG spectral power covariance maps (Gudmundsson et al., 2007).

Another limitation is the invasiveness of the treatment method which induces a minor brain lesion during implantation of the NGF-releasing implants. This is reflected in the temporary increase in the CSF neurofilament light chain (NFL) protein at the 3-month follow-up, returning to baseline levels when measured at 12 months (Eriksdotter-Jönhagen et al., 2012). However, there was no correlation between the qEEG parameters and CSF NFL levels and no significant interaction with time, MMSE, or ChAT.

A possible confounding placebo effect of the implantation procedure is less likely but since it could not be ruled out future investigation requires a cross-validation design.

We have employed only selected conventional EEG parameters that have shown in previous studies reliable test-retest reliability (Gudmundsson et al., 2007; Näpflin et al., 2007) at the individual level, which was important for the present study with the limited number of patients. Since the molecular family of NGFs plays an important role in synaptic plasticity (Liu et al., 2014; Numakawa and Odaka, 2021) it would be of interest to further explore on a larger sample size EEG connectivity measures that are more closely related to the efficacy of neuronal networks.

Although this study is exploratory, the results give further support for the qEEG method as a possible marker of disease activity. Furthermore, it is a desirable outcome measure in clinical trials monitoring treatment efficacy over time since it does not put limits on serial recordings. As with many other treatment strategies in AD, there will probably always be responders and non-responders to therapy and the corresponding need to define these patients with an objective and validated functional measures. The present results motivate the use of qEEG as one of the outcome measures in the clinical trials of novel potential therapeutics in AD. However, future validation studies with different treatment approaches on a larger sample of patients are further warranted.

## Data Availability Statement

The raw data supporting the conclusions of this article will be made available by the authors, without undue reservation, upon request.

## Ethics Statement

Ethical approval was obtained from the Regional Human Ethics Committee of Stockholm. The patients/participants provided their written informed consent to participate in this study.

## Author Contributions

HE, ME, and VJ: conceptualization. TK, PA, BL, GL, LW, TD-S, and VJ: methodology. HE, TK, and VJ: formal analysis. HE, AK, and ME: investigation. HE, ÅS, ME, and VJ: resources. HE and VJ: writing—original draft preparation. TK and VJ: visualisation. ÅS, ME, and VJ: supervision. ME: funding acquisition. All authors have been engaged in review and editing and have read and agreed to the published version of the manuscript.

## Conflict of Interest

LW is an employee of NsGene and owns shares in the company. The remaining authors declare that the research was conducted in the absence of any commercial or financial relationships that could be construed as a potential conflict of interest.

## Publisher’s Note

All claims expressed in this article are solely those of the authors and do not necessarily represent those of their affiliated organizations, or those of the publisher, the editors and the reviewers. Any product that may be evaluated in this article, or claim that may be made by its manufacturer, is not guaranteed or endorsed by the publisher.
